# Long-range wetting transparency on top of layered metal-dielectric substrates

**DOI:** 10.1038/srep27834

**Published:** 2016-06-21

**Authors:** M. A. Noginov, Yuri A. Barnakov, Vladimir Liberman, Srujana Prayakarao, Carl E. Bonner, Evgenii E. Narimanov

**Affiliations:** 1 Center for Materials Research, Norfolk State University, Norfolk, VA 23504, USA; 2 MIT Lincoln Laboratory, 244 Wood Street, Lexington, MA 02420, USA; 3 Birck Nanotechnology Center, Department of Electrical and Computer Engineering, Purdue University, West Lafayette, IN 47907, USA.

## Abstract

It has been recently shown that scores of physical and chemical phenomena (including spontaneous emission, scattering and Förster energy transfer) can be controlled by nonlocal dielectric environments provided by metamaterials with hyperbolic dispersion and simpler metal/dielectric structures. At this time, we have researched van der Waals interactions and experimentally studied wetting of several metallic, dielectric and composite multilayered substrates. We have found that the wetting angle of water on top of MgF_2_ is highly sensitive to the thickness of the MgF_2_ layer and the nature of the underlying substrate that could be positioned as far as ~100 nm beneath the water/MgF_2_ interface. We refer to this phenomenon as long range wetting transparency. The latter effect cannot be described in terms of the most basic model of dispersion van der Waals-London forces based on pair-wise summation of dipole-dipole interactions across an interface or a gap separating the two media. We infer that the experimentally observed gradual change of the wetting angle with increase of the thickness of the MgF_2_ layer can possibly be explained by the distance dependence of the Hamaker function (describing the strength of interaction), which originates from retardation of electromagnetic waves at the distances comparable to a wavelength.

## Introduction

### Effects of non-local dielectric environments

Many processes in quantum and classical physics, involving electronic interactions and transitions in a broad range of frequencies, depend not only on the local dielectric permittivities but also on the dielectric permittivities in physical locations, which can be separated from the point of interest by a distance of multiple wavelengths. (In this paper, we limit our discussion to non-magnetic media.) Thus, it is well known that spontaneous emission can be controlled by the vicinity of a mirror^1^, plasmonic nanostructure^2^ or inside a cavity^3^. Of particular significance are recently explored engineered composite materials (metamaterials) with hyperbolic dispersion^4^,^5^,^6^,^7^ that have a broadband singularity of the (high) photonic density of states^8^ and can control the rate^8^,^9^,^10^,^11^,^12^,^13^, the directionality^8^,^14^ and the spectra^13^,^15^,^16^ of emission of molecules and quantum dots. The other processes, which can be controlled by the vicinity of metamaterials and metallic surfaces and which do not always belong to the traditional domain of electrodynamics include reflection^17^,^18^, stimulated emission^19^, Förster energy transfer^20^, and chemical reactions^21^.

We infer that van der Waals interactions, which determine scores of physical phenomena ranging from phase transitions to friction and wetting, are sensitive to non-local dielectric environments as well. The experimental proof of this heuristic prediction and the observation of the long range wetting transparency are the central results of this paper.

### Van der Waals interactions and dispersion forces

The concept of interaction between neutral atoms or molecules has been proposed by van der Waals in 1873^22^. Subsequently, three different, although related, processes, which contribute to this phenomenon, have been identified as (1) interaction between randomly oriented permanent dipoles and molecules (orientation or Keesom mechanism^23^,^24^,^25^,^26^), (2) interaction between randomly oriented permanent dipoles and induced dipoles (induction or Debye mechanism^27^,^28^), and (3) fluctuating dipole – induced dipole interactions (dispersive or London mechanism^29^.) In the latter case, fluctuation of dipole moments results from thermal or quantum effects. The dispersive mechanism is better studied in the literature and believed to be predominant for interaction between various macroscopic bodies^30^.

**Figure 1:**
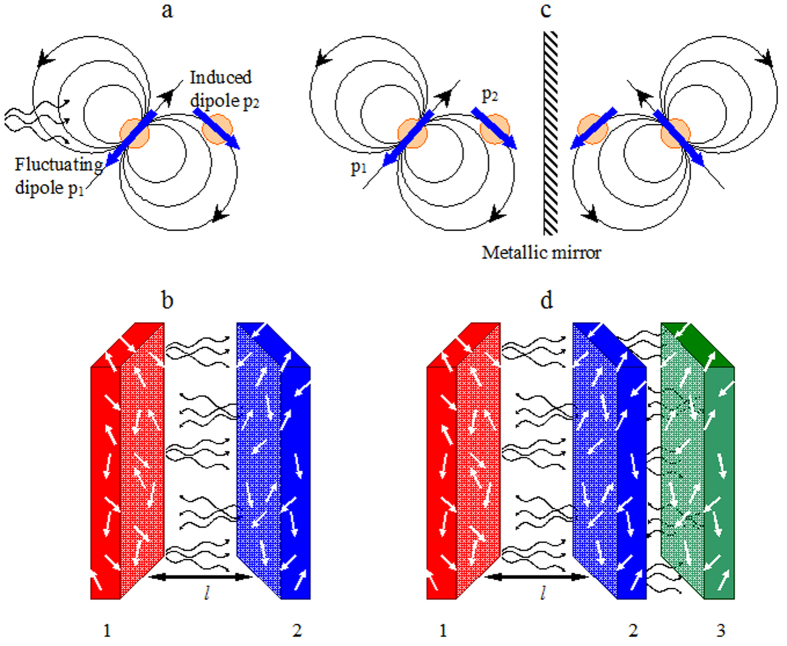
(**a**) Interaction of fluctuating dipole p_1_ and induced dipole p_2_. (**b**) Interaction of two slabs 1 and 2, when the gap between the slabs is filled with the medium M. (**c**) Same as in Fig. 1a in the vicinity of the metallic mirror that produces image charges. (**d**) Same as in Fig. 1b in presence of slab 3. Adopted and adapted from ref. 31.

In Fig. 1a, fluctuating dipole in atom 1 induces dipole in atom 2 and these two dipoles attract each other. If two slabs comprise multiple dipoles, the slabs can be attracted to each other as well, Fig. 1b. The simplest pair-wise summation model assumes the dipoles to be in vacuum and neglects many-body interactions of dipoles within the same slab as well as in different slabs^30^,^31^,^32^,^33^. At the same time, the gap between the slabs can be filled with a medium M, Fig. 1b. This can change both the magnitude and the sign of the force exerted by slabs 1 and 2 on each other^34^. If an external body, such as metallic mirror, is brought to the vicinity of the two dipoles, it will produce virtual image dipoles and alter the net attractive force between atoms 1 and 2, Fig. 1c. Likewise, the external body 3 will change the interaction force between the two macroscopic slabs 1 and 2, Fig. 1d.

Lifshits^34^ and Dzyaloshinskii, Lifshitz and Pitaevskii^35^ have shown that the force *F* between two semi-infinite slabs 1 and 2 separated by the spacer M and the corresponding energy of interaction *U* depend on the dielectric permittivities of all three media involved, *ε*_*1*_(*ω*), *ε*_*2*_(*ω*), *ε*_*M*_(*ω*), integrated over the whole frequency range. In the non-retarded regime, when the distance between the two slabs *l* is much smaller than the speed of light *c* divided by the maximal characteristic frequency *ω*_*UV*_*/*2*π* (of e.g. ultraviolet absorption band) contributing to the interaction energy, the latter is equal to 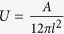. Here *A*, historically termed “Hamaker constant”, is determined by dielectric permittivities or molecular polarizabilities of the interacting media^30^,^32^,^33^. In the simplest case of two identical dielectric slabs separated by vacuum, an approximate value of the Hamaker constant is given by the equation^33^


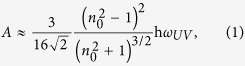


where *n*_*0*_ is the refractive index in the visible range and *ω*_*UV*_ is the characteristic electron polarization angular frequency in the ultraviolet range of the spectrum. In the retarded regime, *l* ≥ 2*πc*/*ω*_*UV*_, the ~*l*^*−*2^ dependence changes to the ~*l*^*−*3^ dependence^30^,^31^,^32^,^33^,^34^,^35^,^36^,^37^. Correspondingly, the largest contribution to the interaction comes from the layers of interfacing media separated by the minimal distance *l*_*0*_ at which the model is still applicable (not less than the molecular size) and the contribution from the layers separated by the distance *xl* becomes *x*^2^ times less important.

Note that the ~*l*^*−*2^ dependence of the interaction energy has a substantially longer range than the ~*l*^*−*6^ dependence for interacting atomic dipoles. This is the important result of Hamaker who has shown (for spheres, in the pair-wise summation approximation) that although the range of atomic forces is of the order of atomic dimensions, the sum of the dispersion energies leads to interaction of nanoscopic colloidal bodies at the characteristic distances comparable to their dimensions^32^,^38^. As was shown later^32^, the Hamaker parameter A may depend on *l* (therefore, “Hamaker function” rather than “Hamaker constant”). This dependence can, in principle, make the range of interaction even longer than *U*~*l*^*−*2^.

### Wetting

**Figure 2:**
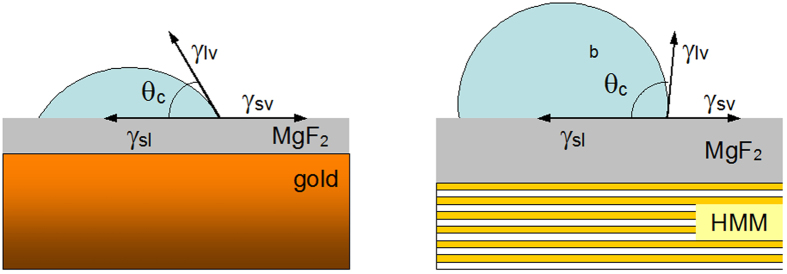
Wetting angle for a droplet of water on top of MgF_2_ layer with gold (**a**) and lamellar matamaterial (**b**) underneath.

The considerations above apply to both solids and liquids. If two interacting media are the same and the spacer is vacuum, then, assuming that the *U*~*l*^*−*2^ model is applicable down to the critical cutoff distance equal to the molecular size *l*_0_^33^, one can calculate the free energy change due to cohesion of the material in vacuum *ΔU*^30^. The latter quantity is related to the surface tension *γ* = *ΔU/*2, *e.g.* of a droplet of liquid in vacuum. If the droplet resides on a solid surface, then, in a similar way, one can introduce the free energy and the surface tension for the liquid-solid interface, *γ*_*ls*_, liquid-vapor interface, *γ*_*lv*_, and solid-liquid interface, *γ*_*sv*_. They collectively determine the wetting angle *θ*, defined as shown in Fig. 2,


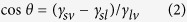


(Young equation^33^,^39^). As we have argued above, the third body brought to the vicinity of slabs 1 and 2 can change the interaction energy between them, Fig. 1d. Correspondingly, we infer that the wetting angle, which can be measured experimentally, should be sensitive to a change of the (non-local) dielectric environment in the vicinity of the solid-liquid interface.

## Experiment

### Idea of the experiment

The van der Waals forces and, in particular, the dispersive London force, are not the only contributors to the surface energy and the wetting angle. Other important contributions come from e.g. the material-specific surface energy determined by polarization and chemical activity of the top molecular layer of the substrate^33^,^40^. Therefore, we designed a series of wetting experiments in which the topmost layer of the solid-state substrate, which was in direct contact with the water droplet, was kept the same, and the underlying medium as well as the corresponding distribution of non-local dielectric permittivity was changed in a systematic manner, Fig. 2. The latter included the change of the thickness of the topmost film.

### Experimental samples and measurements

Three classes of substrates used in this study included glass, Au films, and Au/MgF_2_ lamellar metamaterials – with and without MgF_2_ films deposited on top. Gold films, MgF_2_ films and lamellar Au/MgF_2_ metamaterial samples were fabricated using the thermal vapor deposition technique as described in Methods.

The first series of samples was fabricated by depositing MgF_2_ films of different thickness onto glass substrates. Glass with no MgF_2_ corresponded to zero MgF_2_ film thickness. As we were interested in large wetting angles of water on pristine glass, no special surface treatment, leading to reduction of the wetting angle, was performed^41^.

In the second series of samples, Au films were deposited on glass, after which the MgF_2_ films were deposited on top of gold, Fig. 2a. In 25 samples studied, the thickness of Au layers varied between 25 nm and 150 nm, and the thickness of MgF_2_ layers ranged from ~5 nm to 300 nm, without any intentional correlation between the thicknesses of the metallic and the dielectric films. Several Au films did not have MgF_2_ film on top (zero thickness of MgF_2_).

The third series of samples consisted of four Au/MgF_2_ lamellar metamaterials, Fig. 2b. Each of them had seven alternating Au and MgF_2_ layers, with gold layers on the top and on the bottom. The thicknesses of the layers, the Au filling factors as well as the effective dielectric permittivities in the directions parallel and perpendicular to the layers are discussed in Methods.

In the wetting angle measurements, a droplet of deionized water was placed on top of each fabricated substrate and the wetting angle was measured in the sessile drop experiment^40^,^41^,^42^ as explained in Methods.

### Results

#### MgF_2_ on glass

**Figure 3:**
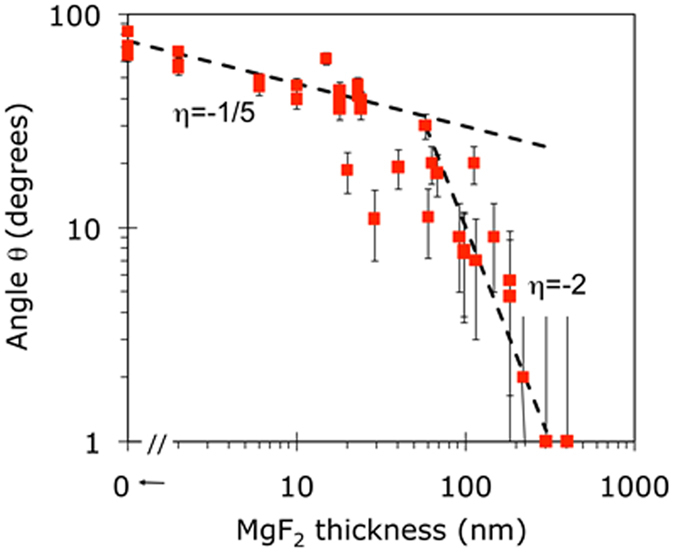
Dependence of the wetting angle on the thickness of the MgF_2_ film deposited on glass. Slopes η = −1/5 and η = −2 are shown as guides for eye. Vertical error bars (±4 degrees) are shown. Horizontal error bars are smaller than the size of the characters.

The results of the first series of experiments are depicted in Fig. 3. At relatively small thicknesses of MgF_2_ films, *l* ≤ 25 nm, the dependence of the contact angle *θ* on *l*, plotted in double logarithmic coordinates, has a fairly small slope *θ*~*l*^*−*1/5^ over first ~25 nm (*θ* changes very slowly with increase of *l*). This relatively small change of the wetting angle is regarded as the *wetting transparency* of the MgF_2_ layer. However, at larger values of *l*, the slope increases dramatically and reaches *θ*~*l*^*−*2^ at *l* ≥ 60 nm. At the thickness of the MgF_2_ film exceeding *l*~200 nm, the wetting angle decreased by more than an order of magnitude and a nearly complete wetting condition, *θ*≈0, has been achieved. Another important observation is that the wetting angle did not experience any noticeable discontinuity as a very thin MgF_2_ film was deposited on top of pristine glass (transition from *l* = 0 to *l* > 0). The latter observation suggests that the wetting angle was not highly sensitive to the chemical composition and corresponding surface energy of the immediate substrate. Instead, the predominant contribution to the wetting angle was coming from long-range forces. As we will see below, both trends (wetting transparency and lack of discontinuity at deposition of thin MgF_2_ layer onto underlying substrate) remain qualitatively unchanged in the case of Au and lamellar metamaterial substrates.

#### MgF_2_ on gold

**Figure 4:**
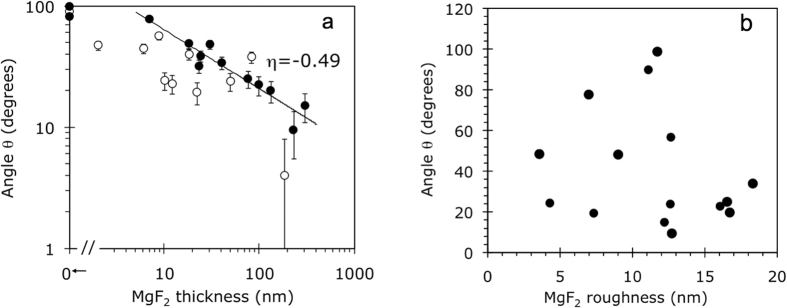
(**a**) Wetting angles measured on top of MgF_2_ films of different thickness deposited on thin (closed circles, average thickness 51 nm) and thick (open circles, average thickness 103 nm) Au films. Vertical error bars (±4 degrees) are shown. Horizontal error bars are smaller than the size of the characters. (**b**) Wetting angles measured on top of MgF_2_ films deposited on Au films (of different thicknesses) as the function of the surface roughness.

The experimental dependence of the contact angle *θ* on the thickness of the MgF_2_ film *l* separating water from gold is shown in Fig. 4a. In line with the discussion above, one can see that as macroscopically thin but microscopically thick layers of MgF_2_ have been deposited on gold, the wetting angle did not change abruptly from the one corresponding to Au (*θ*~90^o^) to the one corresponding to bulk MgF_2_ (*θ*  ≤ 5^o^, nearly complete wetting), which suggests insignificance of the chemical nature of the substrate (in accord with the observation discussed above). Instead, it changed gradually over tens-to-hundreds of nanometers, manifesting strong wetting transparency. This behavior is clearly seen in 13 samples, which had relatively thin Au films (ranging from 25 nm to 73 nm, average thickness 51 nm). The experimental dependence log(*θ*) *vs* log(*l*) is nearly linear, with the slope *η* = −0.49, suggesting the dependence of the contact angle on the thickness of the MgF_2_ spacer 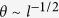.

In the other 12 samples, which had thicker Au films (ranging from 83 nm to 150 nm, average thickness 103 nm), the contact angles, for the same values of *l*, were smaller than those in thin Au film samples, suggesting a somewhat smaller wetting transparency, Fig. 4a. The latter series has also a larger scatter of the data points.

It is important to note that the reduction of the contact angle *θ* with the increase of *l* was not due to larger surface roughness in thicker MgF_2_ films (Wenzel effect [33]). In fact, as the dependence of *θ* on *l* is obvious in Fig. 4a, no dependence of the contact angle on the surface roughness is seen in Fig. 4b.

#### MgF_2_ on lamellar metamaterials

**Figure 5:**
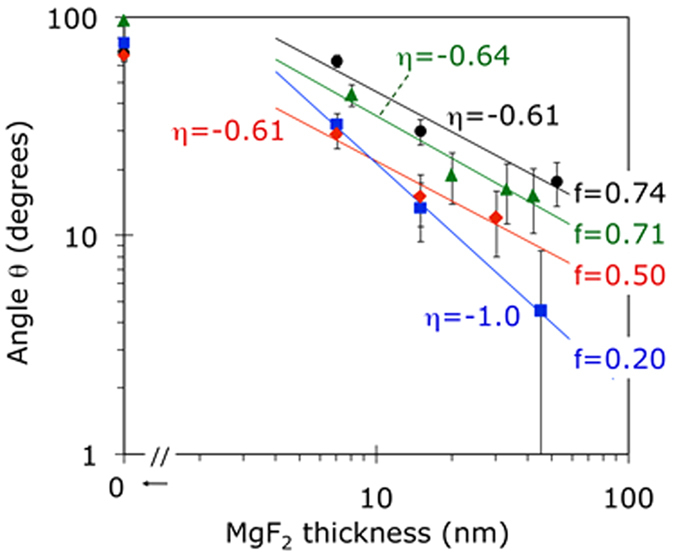
Wetting angles measured on top of MgF_2_ films of different thickness deposited onto lamellar Au/MgF_2_ metamaterial samples, whose metal filling factors are equal to f = 74% in Sample MM1, f = 71% in Sample MM2, f = 50% in Sample MM3, and f = 20% in Sample MM4 (see the sample descriptions in the text). Vertical error bars (±4 degrees) are shown. Horizontal error bars are smaller than the size of the characters.

As discussed above, the topmost layer of lamellar metamaterials used in our studies was gold. Experimentally, we first measured the contact angle of water on top of the upper Au layer of a pristine metamaterial. Then we dried the sample, deposited a thin film of MgF_2_ on its top and repeated the contact angle measurement. After that, the cycle (deposition of another layer of MgF_2_ and contact angle measurement) was repeated multiple times. The results of these measurements are summarized in Fig. 5. One can see that the dependence of the contact angle *θ* on thickness of the MgF_2_ spacer *l*, measured on top of metamaterials, is qualitatively similar to that on top of single gold films, Fig. 4. However, the slopes of the functions *θ*(*λ*) (plotted in double logarithmic coordinates) were larger in the metamaterials samples than in the Au film samples.

The wetting transparency seen in Fig. 5 is getting stronger (the effect of the metamaterial substrate on the wetting angle is observed at larger distances) with increase of the Au filling factor in the metamaterial. At first glance, this appears to be a simple effect of the overall amount of gold: the larger the filling factor of Au, the larger its long-distance effect on the wetting angle. However, this simple reasoning does not hold for wetting angles measured on top of thin and thick Au films, Fig. 4a.

### Synopsis of the experimental observations

The results of the experimental studies can be summarized as follows:

- The wetting angle of water on top of thick (*l* ≥ 100 nm to *l* ≥ 400 nm) layer of MgF_2_ is small (*θ* ≤ 10^o^), approximating the condition of total wetting (*θ* → 0).
- The latter wetting angle is substantially smaller than those measured on top of untreated glass (65^o^ to 85^o^), Au films (80^o^ to 100^o^) and Au/MgF_2_ metamaterials (65^o^ to 95^o^).
- As the thin layer of MgF_2_ is deposited on top of pristine glass, gold or lamellar metamaterial, the wetting angle does not change abruptly from the one measured on top of the original underlying substrate ( ≥ 65^o^) to the one characteristic of thick MgF_2_ ( ≤ 10^o^). Instead, it changes between the two limits (*l* → 0 and *l* → ∞) gradually and smoothly.
- The effect of the *underlying* glass, gold or metamaterial substrate on the wetting angle remains pronounced for MgF_2_ layer thickness as large as several tens or even hundreds of nanometers.

Therefore, we conclude that the chemical nature of the immediate substrate and the chemical activity of its surface do not play a detrimental role in defining the wetting angle, whereas the wetting angle is highly sensitive to the nonlocal dielectric environment beneath the liquid/solid interface.

## Discussion

An important question arises: what kind of dependence of the wetting angle *θ* on the thickness of the MgF_2_ film *l* should be expected and whether our experimental results agree with the theoretical predictions.

The effect of gradual change of the wetting angle with increase of the thickness of the immediate underlying substrate (like MgF_2_ in our experiment) is known in the literature as wetting transparency^41^. Thus, in ref. 41, the wetting angle of water on top of one, two or three layers of graphene was the same or almost the same as that on top of underlying Au, Si, and Cu substrates. The dependence of *θ* on *l* was related to the distance dependence of the interfacial energy *W*(*h*) and the work of adhesion *W*_*a*_ *via* the Young–Dupre equation


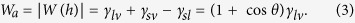


Assuming the 12-6 Lennard-Jones form of the van der Waals interaction, the interfacial energy *W*(*h*) was described as^41^


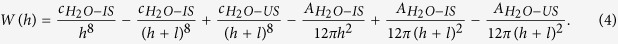


Here, the coefficients *c*_*i−j*_ denote the strength of short-range repulsion, Hamaker constants *A*_*i-j*_ quantify van der Waals attraction between media i and j, *h* is the minimal equilibrium distance between water and the interfacing medium (of the order of the molecular size), *l* is the thickness of the *immediate substrate* (IS: graphene in ref. 41 and MgF_2_ in our work), and US stands for *underlying substrate* (copper in ref. 41 and glass, Au or metamaterial in our case). In accord with the arguments discussed in the beginning of this paper, the wan der Waals attraction terms in Eq. 4 have inverse quadratic dependence on the distance between two flat interfaces. In ref. 40, the equation of a similar structure, although for *spreading* defined as 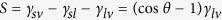
33,40, was used to describe wetting transparency of polystyrene on Si substrate.

For realistic values of coefficients c_*i−j*_and *A*_*i−j*_, and distances *h* ranging between 0.15 nm and 0.20 nm^33^, Eqs. 2 and 3 predict strong dependence of the work of adhesion *W*_*a*_ and the contact angle *θ* on the thickness of the immediate substrate *l* at *l* ≈ *h* and almost complete independence of *θ* of *l* at *l* ≥ 10 *h*. Correspondingly, this model can adequately predict wetting transparency of few 0.3 nm layers of graphene^41^ and completely fails to describe gradual change of the contact angle occurring over ~100 nm distances observed in polysterene on top of Si^40^ or in our experiments reported above.

As the experimentally observed dependence *θ*(*l*) cannot be explained by the simple model based on summation of all pair-wise van der Waals interactions across the interface, one should look for alternative explanations. In ref. 40, the gradual increase of the wetting angle with increase of the thickness of the polystyrene film *l* (that was in direct contact with a droplet of water) was explained by the thickness dependence of the polystyrene’s refractive index *n*. The latter parameter determines the Hamaker constant *A* (Equation 1) and, in line with Eqs. 3 and 4, controls the work of adhesion *W*_*a*_ and the wetting angle *θ*. In ref. 40, an increase of the thickness of the polystyrene film thickness from 21 nm to 625 nm caused *Δn* = 5% reduction of the refractive index and *ΔA* = 26% reduction of the Hamaker constant, which led to increase of the wetting angle from 81.3^o^ to 86.6^o^.

**Figure 6:**
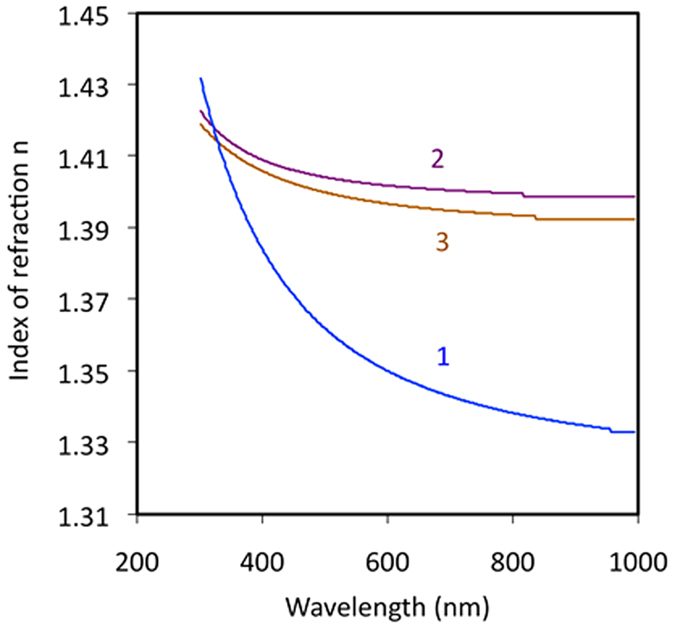
Spectra of the index of refraction in MgF_2_ films of different thickness l deposited on glass. (1) l = 37 nm, (2) l = 165 nm, (3) l = 346 nm.

In order to evaluate the effect of the MgF_2_ refractive index on the wetting angle in our experiments, we have performed ellipsomerty measurements on several MgF_2_ films of different thickness *l* deposited on top of thick (200 nm) Au films on glass, See Methods. The representative spectra of the experimentally obtained refractive indexes *n* (which are within the range of data available in the literature^43^,^44^,^45^) are shown in Fig. 6. The values of *n* are nearly independent of the film thickness at *l* ≥ 150 nm (the trend shared by other thick MgF_2_ films) and decrease at *l* = 28 nm, Fig. 6. One can see that at *λ* = 600, with increase of the film thickness from 28 nm to *e.g.* 165 nm, the refractive index increases by 3.7% and the corresponding Hamaker constant, calculated according to Eq. 1, increases by 22%. Therefore, the qualitative trend observed in our experiment – increase of *n* and *A* corresponding to the reduction of the contact angle *θ* – is the same as in ref. 40. However, no quantitative evaluation of the effect of *n* on the contact angle *θ* is possible, in particular, because the wavelength, at which *n* should be evaluated (in Equation 1) is not well defined and *Δn* diminishes toward shorter wavelengths and even changes sign at *λ* < 320 nm. Thus, although the change of *n* with increase of the MgF_2_ film thickness *l* can contribute to the observed reduction of the contact angle *θ*, it remains questionable whether this effect is strong enough to fully account for the nearly one order of magnitude decrease of *θ* in our experiments.

The very strong dependence of the wetting angle on the thickness of highly viscoelastic films observed in ref. 42 was explained by the films deformation caused by surface tension forces. This explanation is limited to soft matter and unlikely can be extended to rigid MgF_2_ films used in our experiments.

In further search for possible explanations of the wetting transparency observed in our experiments, we can recall that water molecules are highly polar and that the dispersion force contribution to the overall van der Waals energy of two interacting water molecules is only 24%^33^. (This percentage is higher for interaction of water molecules with many dissimilar molecules, e.g. 87% for interaction of HO_2_ and CH_4_^33^.) This fact, in combination with hypothetic surface charges of the topmost and/or underlying solid state layers, could, in principle, lead to deviation from the *l*^−2^ dependence for the interaction energy of two slabs and cause long-range sensitivity of the wetting angle to the existence of the underlying substrate separated from water by tens-to-hundreds of nanometers of MgF_2_ spacer. However, as we have experimentally demonstrated insensitivity of the wetting angle to the chemical nature of the immediate topmost substrate (which is in direct contact with water), this explanation appears to be unlikely as well.

We lastly recollect that the strength of interaction of the two slabs separated by a spacer depends on spectroscopic properties of all three media involved and the spectral positions of their polarization (absorption) bands, which make predominant contributions to the dispersion forces. Depending on the spectroscopic details, it is even possible for the van der Waals-London interaction between two different materials immersed in a liquid to be repulsive^30^,^32^. Large separation between the two slabs, of the order of characteristic electromagnetic wavelength, causes wave retardation and reduces the force and the energy of the interaction. As the distance *l* between the two slabs is gradually increased, the retardation first occurs for high frequency polarization components and then progresses toward lower frequencies. As a result, the effective Hamaker constant (now Hamaker function) can change not only its value, but even the sign, as the separation distance *l* between the two slabs gradually increases. Therefore, the situations are possible where the “attractive” forces, which are attractive at long distances, become repulsive at intermediate distances and change back to attractive at even smaller values of *l* ref. 32. Such a strong distance dependence of the Hamaker function can overcome the *l*^−2^ scaling of the interaction energy between the two slabs and, potentially, explain anomalous long-range wetting transparency observed in our experiments. Detailed study of this phenomenon is a subject of future work.

Note that although the slope of the {log_10_(*θ*) vs log_10_(*l*)} dependence was different in the Au films and Au/MgF_2_ lamellar samples, this disparity could not be related to the metamaterials’ hyperbolic dispersion.

## Summary

We have experimentally studied wetting of several metallic, dielectric and lamellar metal/dielectric substrates, including glass, thin and thick Au films and Au/MgF_2_ stacks – both pristine and coated with MgF_2_ layers of varied thickness. We have found that the wetting angle on top of thick, *l* ≥ 100 nm to *l* ≥ 400 nm, MgF_2_ layers is  ≤ 10^o^, approximating the total wetting condition. At the same time, wetting angles on top of pristine glass, Au films and Au/MgF_2_ metamaterials (with Au outmost layer) are significantly larger,  ≥ 65^o^. As thin layers of MgF_2_ are deposited on top of glass, Au or Au/MgF_2_ metamaterial, the contact angle does not change abruptly from the one corresponding to the underlying substrate to the one corresponding to thick MgF_*2*_. We, thus, conclude that the wetting angle is nearly insensitive to the chemical nature of the immediate substrate and its surface. Instead, as the thickness of the MgF_2_ layer is gradually increased from *l* = 0 nm (no MgF_2_) to *l* > 100 nm, the wetting angle changes smoothly and continuously, demonstrating sensitivity to the presence of underlying substrate at the thickness of the MgF_2_ layer of the order of ~100 nm. We regard this phenomenon as long-range wetting transparency.

The semi-qualitative analysis suggests that if the interaction energy of two parallel slabs changes with the distance as *l*^−2^ (the behavior predicted by the most simple model of dispersion van der Waals interactions), the wetting transparency, although possible, should not extend longer than several nanometers. The gradual change of the wetting angle occurring over tens of nanometers of the MgF_2_ spacer can be partly due to the thickness dependence of the MgF_2_ refractive index. However, it is unlikely that this effect can fully account for the whole magnitude of observed phenomenon (change of *θ* from from  ≥ 65^o^ to  ≤ 10^o^). We infer that the experimentally observed long-range wetting transparency can possibly be due to strong distance dependence of the Hamaker function, which determines the strength of the dispersion van der Waals- London interactions, related to retardation of electromagnetic radiation.

## Methods

### Thin film deposition

Gold films, MgF_2_ films and lamellar Au/MgF_2_ metamaterial samples were fabricated using the thermal vapor deposition technique (in the Edwards BOC Auto-306 thermal evaporator) at vacuum better than 10^−6^ mbar. All films and multi-layered stacks were deposited onto glass substrates (silica-based Micro cover glass, 22 mm × 22 mm from VWR (48366-067), index of refraction n = 1.517 at *λ* = 546.07 nm). The thickness of the thin films as well as the metamaterial stacks was evaluated by making a scratch and measuring its profile with the atomic force microscope (AFM, Multiview-4000 from Nanonics Imaging). The results were averaged over several measurements performed on the same sample. The evaluated surface roughness (thickness inhomogeneity) was ~5 nm in thin layers (*d* ≤ 20 nm) and could be even larger in thicker MgF_2_ films (*d* ≥ 50 nm).

### Lamellar metamaterial samples

Four Au/MgF_2_ lamellar metamaterial samples have been fabricated using thermal vapor deposition technique as discussed above, Fig. 2b. Each of them had seven alternating Au and MgF_2_ layers, with gold layers on the top and on the bottom. The thicknesses of the layers were as follows: 20 nm thick Au and 7 nm thick MgF_2_ in the sample MM1; 25 nm thick Au and 10 nm thick MgF_2_ in the sample MM2; 20 nm thick Au and 20 nm MgF_2_ in the sample MM3; and 15 nm thick Au and 60 nm thick MgF_2_ in the sample MM4. All samples had gold-like luster appearance, which suggested that Au films were continuous layers rather than ensembles of isolated Au nano-islands, which have dark coloration. Although the thickness inhomogeneity/roughness of the 7 nm thick and 10 nm thick MgF_2_ films was relatively large, the corresponding metamaterial samples followed the general trend seen in Fig. 5 (as discussed in *Experimental results*), which suggests adequate precision of fabrication and thickness measurements.

Based on the effective medium model for dielectric permittivities of stratified media^46^,^47^ and known dielectric permittivities and filling factors of gold^48^ and MgF_2_^43^,^44^, the fabricated metamaterials had hyperbolic dispersion (Re(*ε*_||_) < 0 and Re(*ε*_⊥_) > 0) at *λ* > 490 nm in Sample MM3, and *λ* > 580 nm in Sample MM4. Samples MM1 and MM2 were formally hyperbolic in the whole infrared-to-near ultraviolet range of the spectrum. However, at *λ* ≤ 490 nm, their real parts of *ε*_||_ were negative but substantially small, |Re(*ε*_||_)| < 1. Here *ε*_||_ and *ε*_⊥_ are the effective (real) dielectric permittivities of the lamellar metamaterial in the directions parallel and perpendicular to the metamaterial’s surface, correspondingly. The MgF_2_ films of varied thickness were deposited on top of the metamaterials as discussed in *Experimental results*).

### Wetting angle measurements

In the sessile drop wetting experiment^40^,^41^,^42^, a droplet of deionized water (1 to 2 millimeters in diameter) was placed onto various substrates described above. The advancing (maximal) wetting angle was measured using the contact angle goniometer and high-resolution CCD camera (from Logitech). Several measurements were performed on different parts of each sample, after which the results were averaged. As a rule, the wetting angle measurements were performed on freshly made samples fabricated on the same day. The nominal ambient temperature was 23C. Unintentional variations of the environment, including accidental static charges, modestly affected the value of the measured wetting angle. However, the accuracy of the measurement was always better than ±4 degrees.

### Ellipsometry measurements

Ellipsometric measurements were performed over the wavelength range from 250 nm to 1000 nm and four angles of incidence from 50 to 70 degrees. A control sample of blank Au film was measured and its optical constants were extracted. These Au optical constants were used when modeling MgF_2_/Au film stacks. Since MgF_2_ films were transparent in the measured wavelength range, Cauchy dispersive model was used to extract the index of the MgF_2_ films.

## Additional Information

**How to cite this article**: Noginov, M. A. *et al.* Long-range wetting transparency on top of layered metal-dielectric substrates. *Sci. Rep.* **6**, 27834; doi: 10.1038/srep27834 (2016).
